# Comprehensive Multi-Dimensional MRI for the Simultaneous Assessment of Cardiopulmonary Anatomy and Physiology

**DOI:** 10.1038/s41598-017-04676-8

**Published:** 2017-07-13

**Authors:** Joseph Y. Cheng, Tao Zhang, Marcus T. Alley, Martin Uecker, Michael Lustig, John M. Pauly, Shreyas S. Vasanawala

**Affiliations:** 1 Stanford University, Department of Radiology, Stanford, California, USA; 2 GE Healthcare, Houston, Texas USA; 3 German Center for Cardiovascular Research, Partner Site, Göttingen, Germany; 4 University Medical Center, Department of Diagnostic and Interventional Radiology, Göttingen, Germany; 5 University of California, Berkeley, Department of Electrical Engineering and Computer Sciences, Berkeley, California, USA; 6 Stanford University, Department of Electrical Engineering, Stanford, California, USA

## Abstract

Diagnostic testing often assesses the cardiovascular or respiratory systems in isolation, ignoring the major pathophysiologic interactions between the systems in many diseases. When both systems are assessed currently, multiple modalities are utilized in costly fashion with burdensome logistics and decreased accessibility. Thus, we have developed a new acquisition and reconstruction paradigm using the flexibility of MRI to enable a comprehensive exam from a single 5–15 min scan. We constructed a compressive-sensing approach to pseudo-randomly acquire highly subsampled, multi-dimensionally-encoded and time-stamped data from which we reconstruct volumetric cardiac and respiratory motion phases, contrast-agent dynamics, and blood flow velocity fields. The proposed method, named XD flow, is demonstrated for (a) evaluating congenital heart disease, where the impact of bulk motion is reduced in a non-sedated neonatal patient and (b) where the observation of the impact of respiration on flow is necessary for diagnostics; (c) cardiopulmonary imaging, where cardiovascular flow, function, and anatomy information is needed along with pulmonary perfusion quantification; and in (d) renal function imaging, where blood velocities and glomerular filtration rates are simultaneously measured, which highlights the generality of the technique. XD flow has the ability to improve quantification and to provide additional data for patient diagnosis for comprehensive evaluations.

## Introduction

Pediatric cardiovascular and respiratory diseases, including congenital heart diseases (CHD), lung disease of prematurity, and cystic fibrosis, are common and result in serial imaging studies that assess the cardiovascular and pulmonary systems in isolation. Due to complex coupling between the cardiovascular system and respiratory system, a disease afflicting one system impacts the other^1, 2^. Therefore, better understanding of these disease states and more effective treatment would be enabled by comprehensive assessment of both cardiovascular and respiratory systems together.

Currently, no single comprehensive test exists for cardiopulmonary evaluation. The convention is to use multiple exams with patients in varying physiologic states^3, 4^: a lung scintigraphy scan for ventilation assessment, a perfusion scan with intravenous injection, and a computed tomography or magnetic resonance imaging (MRI) exam for cardiac evaluation. Each exam adds risk to already fragile patients. Moreover, logistics of performing multiple exams increase healthcare costs^5, 6^ and often delay care of patients who may require urgent management. Thus, only a subset of exams is typically performed, and major clinical decisions, such as surgical procedures, are based on incomplete information.

MRI has the potential to provide comprehensive evaluations for anatomy and function of different organ systems. However, the flexibility of this imaging modality increases the complexity of the exam; different techniques are optimized for each specific clinical question^7, 8^. Though simultaneous multi-modal imaging exists, such as MRI with positron emission tomography^9^, these systems further complicate imaging exams. Recently, major efforts have been made to shorten and simplify the MRI protocol to address some of these issues. First, the entire MRI workflow can be simplified and made more efficient with automated protocol parameter optimization^10^. The number of scans required can also be reduced. For example, a single sequence can be optimized^11^ or randomized with MR fingerprinting^12^ to quantify multiple tissue properties (i.e., T1, T2*, proton density) as biomarkers. Additionally, contrast-enhanced volumetric cardiac-resolved flow imaging (4D flow)^13, 14^ has been shown to enable the assessment of flow, function, and anatomy from a single MRI sequence^15–19^. Such a sequence is compelling for many clinical applications including the assessment of CHD in pediatric patients^20^. With current accelerated imaging techniques using parallel imaging and compressed sensing^20–24^, 4D flow with high spatial-temporal resolution is performed in a single 5–15 min scan^25–28^. We hypothesize that these accelerated imaging techniques can not only achieve clinical feasible scan durations but can also be exploited to lift 4D flow imaging to higher-dimensional space for multi-dimensional flow imaging. Conventional 4D flow imaging consists of cardiac-phase dimension, volumetric spatial dimensions, and three-dimensional blood flow velocities. In addition to the dimensions of 4D flow, we will also include respiratory-phase dimension and/or temporal dimension (for contrast enhancement).

The extension of conventional imaging approaches to higher-dimensional space has been recently shown to improve anatomical image quality for MRI^29, 30^. For scan durations of 5–15 min, scans are sensitive to errors introduced from patient motion, from physiological variations, and from changes in the contrast signal for contrast-enhanced scans. Correcting for these different effects can be quite challenging. For example, patient motion not only introduces phase changes in the k-space signal but the movement also changes the underlying B0 field^31, 32^. L. Feng *et al*. proposed to resolve these different dynamics in the MR datasets as additional dimensions in an “extra-dimensional space,” or XD space, instead of explicitly correcting for them^29^. Here, our goal is to not only enable greater tolerance to motion and other previously ignored physiological variations by lifting 4D flow to a higher-dimensional space, but we also aim to enhance the MRI sequence with additional clinical information for a more comprehensive imaging study. Since this work is inspired by reconstructing in the XD space, we refer to the proposed multi-dimensional flow imaging as XD flow.

XD flow enables greater flexibility to probe physiology with MRI. By resolving respiratory motion, flow and function can be assessed for both inspiratory and expiratory respiratory phases^33^. Also, by resolving contrast dynamics, myocardial or lung perfusion can be quantified. By extending 4D flow into higher-dimensional space, the technique can potentially enable comprehensive exams, such as for cardiopulmonary diseases, from a single sequence. The purpose of this work is to develop the framework for transforming 4D flow to XD flow imaging and to demonstrate the feasibility of XD flow in a number of representative use cases for pediatric MRI.

## Results

**Figure 1:**
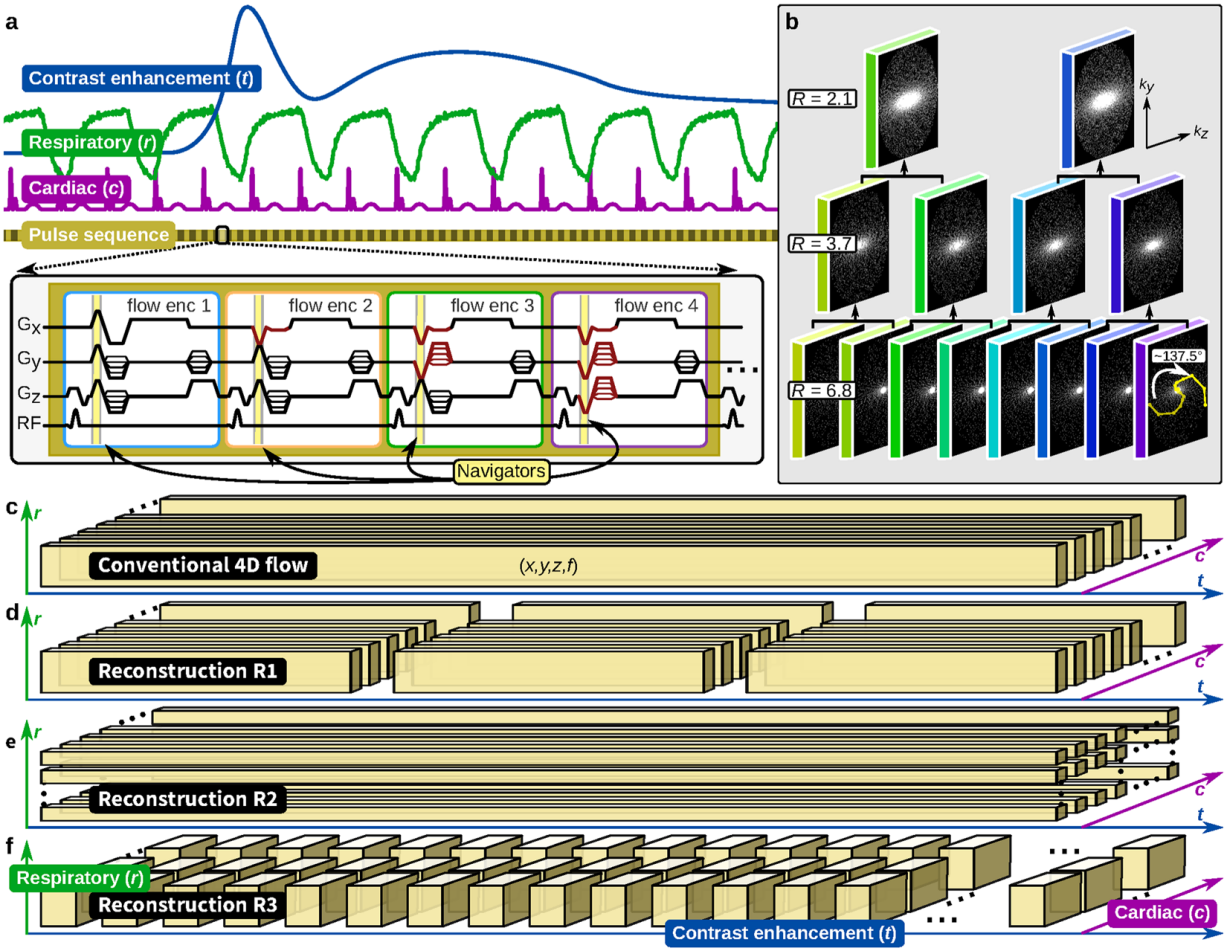
Data acquisition overview. (**a**) Pulse sequence diagram. (**b**) Cartesian sampling masks generated using the variable-density and radial view-ordering (VDRad) design. (**c**–**f**) Single dataset acquired in the multi-dimensional space reconstructed with different methods of data binning where each data block consists of three-dimensional spatial (*x*, *y*, *z*) and flow encoding (*f*). In (**a**) different dimensions are illustrated: dynamic contrast enhancement (*t*), respiratory phase (*r*), and cardiac phase (*c*). For volumetric flow quantification, four different flow-encoding gradients are used to sensitize the acquisition to four different velocities. The flow-encoding gradients provide the setup to intrinsically measure motion using Butterfly navigators. In (**b**) VDRad determines the order in which each (*k*_*y*_, *k*_*z*_)-sample is collected using the golden-ratio angle increment (~137.5°). The same samples can be divided into different number of sampling masks where fewer masks correspond to lower subsampling reduction factors (from *R* of 6.8 to 2.1). This property is important for retrospective binning of the data. The variable-density sampling provides ideal source data for compressed sensing reconstructions. Conventional 4D flow depicted in (**c**) ignores the *r*, *c*, and *t* dimensions. XD flow extends this same single dataset into higher-dimensional-space for highlighting different clinical indications. Three different examples of this flexibility is illustrated in (**d**–**f**).

XD flow covers a multi-dimensional space consisting of three spatial dimensions (*x*, *y*, *z*), one cardiac-phase dimension (*c*), one respiratory-phase dimension (*r*), one temporal dimension (*t*), and velocity-encoding echoes (*f*). To demonstrate the feasibility of XD flow, we projected the dataset into a smaller number of dimensions and performed the reconstruction in these subspaces to highlight different features (Fig. 1):

- *Time-resolved, cardiac-resolved, high-resolution flow imaging in (x, y, z, t, c, f)-space:* The acquisition was binned into 2–4 large temporal windows, and high-resolution cardiac-resolved volumetric flow data were reconstructed. Impact of respiratory motion (*r*) was minimized using soft-gating. This reconstruction focused on minimizing the impact from the contrast dynamics for contrast-enhanced scans and from bulk patient motion for high-resolution cardiovascular imaging (Fig. 1d).

- *Respiratory-resolved*, *cardiac-resolved*, *high-resolution flow imaging in* (*x*, *y*, *z*, *c*, *r*, *f*)*-space*: Cardiac-resolved volumetric flow datasets were reconstructed for each respiratory state. Here, the temporal component (*t*) was ignored. This reconstruction enabled the evaluation of the impact of respiration on cardiac function (Fig. 1e).

- *Cardiac-resolved*, *dynamic-contrast-enhancement* (*DCE*) *or perfusion imaging in* (*x*, *y*, *z*, *t*, *c*)*-space*: For a 1–2 min window during the contrast injection, data were binned into ~2-s temporal windows with few cardiac phases (≤5). A single echo (*f* = 0) was reconstructed with data from other echoes included with an extra weighting factor of 0.5. Impact of respiratory motion (*r*) was minimized using soft-gating. This reconstruction was used to enable perfusion analysis (Fig. 1f).

Implementation details are described in the Methods.

With Institutional Review Board approval and informed consent/assent, pediatric patients referred either for contrast-enhanced 3T MRI or contrast-enhanced 1.5T MRI were recruited and scanned. Specific scan parameters are described in Table 1 and Supplementary Table S1. First, the R1 reconstruction (few temporal phases with many cardiac phases) was performed in a newborn study with the purpose of reducing image artifacts from bulk voluntary motion. The impact of variations in the blood pool signal due to contrast dynamics was emphasized in another study where gadolinium-based contrast was administered during the acquisition, and R1 was performed to reduce image artifacts. The impact of respiration on blood flow was investigated using R2 (respiratory-resolved reconstruction). Lastly, the potential of including image analysis on the contrast dynamics from XD flow using R3 (high temporal resolution with few cardiac phases) was assessed in a cardiac/pulmonary study and in an abdominal study. For comparison in each of these cases, a conventional 4D flow reconstruction^25^ from the same dataset was performed.

**Table 1:** Summary of scan parameters.

	#1	#2	#3	#4	#5	#6
	(Fig. 2)	(Fig. 4)	(Fig. 5)	(Fig. 6)	(Fig. 7)	(Fig. 8)
Age	3 days	11 months	3 years	2 years	8 years	15 years
Gender	F	F	F	M	M	M
Heart rate	135 bpm	111 bpm	107 bpm	75 bpm	98 bpm	90 bpm
TE/TR	1.7 ms/4.2 ms	1.8 ms/4.0 ms	1.8 ms/4.0 ms	1.7 ms/5.8 ms	1.7 ms/4.8 ms	1.8 ms/4.0 ms
Resolution	(0.9, 0.8, 1.4) mm	(1.0, 0.9, 1.4) mm	(1.0, 0.7, 1.4) mm	(2.1, 2.0, 3.0) mm	(2.0, 1.9, 2.4) mm	(1.7, 1.5, 2.0) mm
Bandwidth	±125 kHz	±83.33 kHz	±83.33 kHz	±100 kHz	±125 kHz	±83.33 kHz
VENC	250 cm/s	250 cm/s	250 cm/s	250 cm/s	100 cm/s	150 cm/s
Coil	32ch cardiac	32ch cardiac	32ch cardiac	20ch chest	32ch body	32ch body
Contrast	Ferumoxytol	Gadofosveset trisodium	Ferumoxytol	Gadobutrol	Gadobutrol	Gadofosveset trisodium
Scan time	10:34 min	8:23 min	10:05 min	9:30 min	7:14 min	7:22 min
Scanner	3T (GE MR750)	3T (GE MR750)	3T (GE MR 750)	1.5T (GE 450 W)	3T (GE MR750)	3T (GE MR 750)

### Comprehensive congenital-heart-disease MRI exam

**Figure 2:**
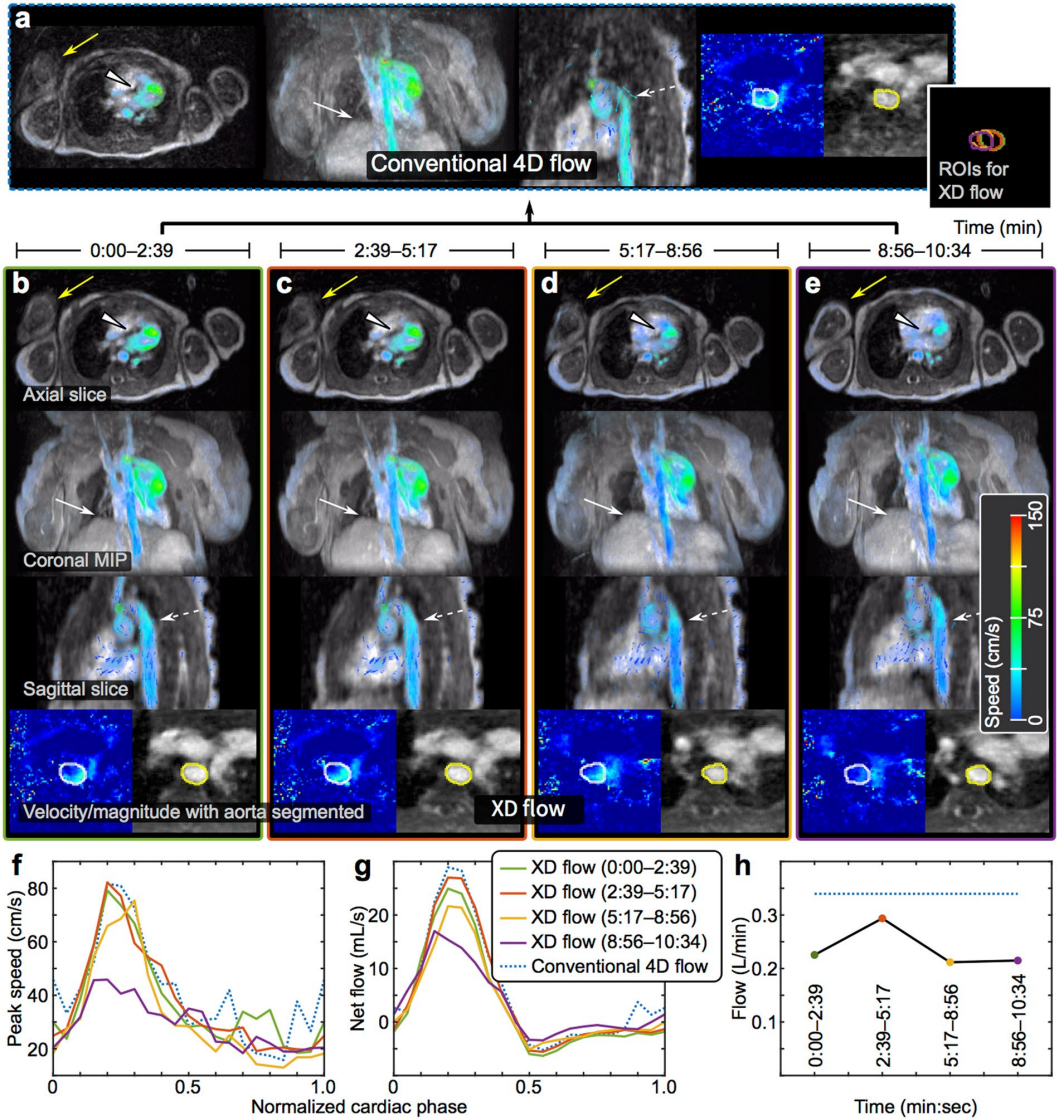
XD flow reconstruction of a 3-day-old female highlighting enhanced motion robustness. (**a**) Conventional 4D flow reconstruction for the 10:34 min acquisition. (**b**–**e**) The same dataset reconstructed into 4 shorter temporal windows — each window was 2:39 min. (**f**) Peak speed in aorta. (**g**) Net flow in aorta. (**h**) Average flow for each temporal window compared to conventional 4D flow (dotted blue). Different reformats of a single cardiac phase are shown: axial slice, coronal 50-cm MIP, sagittal slice, and velocity/magnitude of aorta with segmentation. The non-sedated patient with ferumoxytol enhancement was observed to be moving as noted by different positions of the right arm (yellow arrow). Also, the region-of-interests (ROIs) segmented for each temporal phase of XD flow are combined to emphasize the movement of the aorta (far right of (**a**)). With motion corruption, the flow in the aorta is noisy with unrealizable flow vectors in the conventional 4D flow, but the flow is recovered in the XD flow reconstruction (dashed white). Similarly, the myocardial border (white triangle) and diaphragm (white arrow) are better depicted in the XD flow reconstruction. The blood flow measured from XD flow varies over time; this effect is ignored in conventional 4D flow.

**Figure 3:**
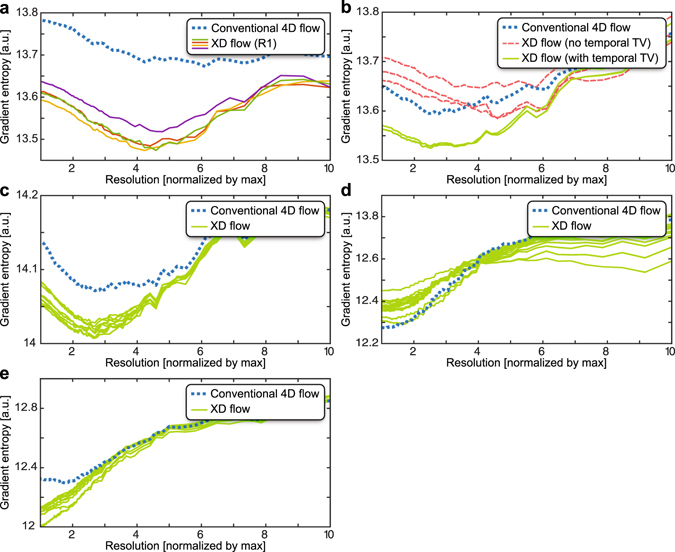
Image sharpness computed using the gradient entropy metric^49^ (lower values means sharper images). The spatial resolutions of the final reconstructed images were retrospectively lowered, and this metric was computed as a function of image resolution. In the plots, the resolution is normalized by the maximum resolution — 1 corresponds to the acquired spatial resolution, 2 corresponds to 2x lower spatial resolutions, and so on. The true underlying spatial resolution can be considered as the minimum of the curve. This analysis was performed for each XD flow dataset (green or with corresponding colors of the associated figure) and was compared to the original conventional 4D flow reconstruction (dotted blue): (**a**) subject #1 (Fig. 2) (**b**) subject #2 (Fig. 4) (**c**) subject #3 (Fig. 5) (**d**) subject #4 (Fig. 6), and (**e**) subject #5 (Fig. 7). Compared to conventional 4D flow, sharper images was observed for XD flow that was able to resolve bulk patient motion as plotted in (**a**). In other situations, XD flow resulted in lower image resolutions when resolving high-temporal resolution dynamics compared to conventional 4D flow as plotted in (**d**).

The high-resolution flow reconstruction was assessed in a 3-day-old non-anesthetized female with ferumoxytol administration as shown in Fig. 2. With the risks and complications from using anesthesia^34–36^, we aimed to perform exams for neonatal patients without anesthesia. In the conventional 4D flow reconstruction, patient motion resulted in an increase in apparent noise and signal dropout. The recovery of the patient’s arm and the sharpening of the upper liver dome and myocardial borders can be seen in the XD flow reconstruction. A lower average flow (~0.25 L/min) that varies throughout time can be seen from the XD flow reconstruction compared to the conventional reconstruction (~0.35 L/min). Additionally, by dividing the acquisition into 4 temporal bins versus a single average, a reduction of the velocity standard deviation (from ±6.2 cm/s to ±5.2 cm/s in Supplementary Table S2) and an increase in image sharpness (Fig. 3a) were observed.

**Figure 4:**
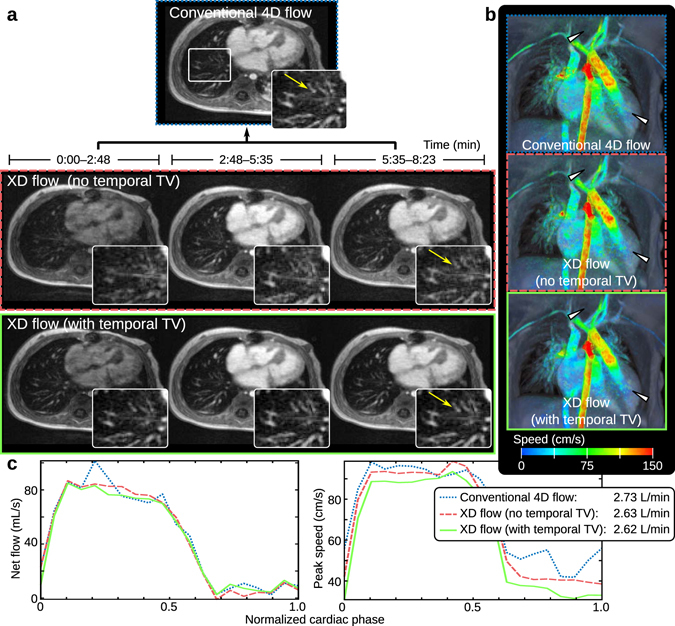
XD flow of a 11-month-old female with reconstruction R1 to minimize the impact of contrast signal fluctuations during the administration of gadofosveset trisodium. In (**a**), the conventional 4D flow of the 8:23 min scan is displayed above the XD flow reconstruction without the temporal total variation constraint (middle) and with the temporal constraint (bottom). The recovery of fine pulmonary vessels (yellow arrow in magnified view) can be seen when exploiting the temporal dimension in the XD flow reconstruction. Surface renderings with velocity overlaid of each reconstruction (last temporal phases for the XD flow reconstructions) are displayed in (**b**). Reduction of noise in the velocity can be observed when comparing the XD flow with conventional 4D flow (white triangles). The lowest noise is observed in the XD flow reconstruction with the temporal total variation constraint. In (**c**), flow (net flow and peak speed) is measured in the aorta root. The average flow of 2.6–2.7 L/min for all three methods are similar.

The impact of variation in contrast dynamics was assessed in a contrast-enhanced study of an 11-month-old female using a gadolinium-based contrast, gadofosveset trisodium. To emphasize change in contrast dynamics, contrast was administered during the data acquisition. Conventional 4D flow reconstruction and two different variations of R1 (with 3 temporal bins) were performed. One R1 was performed without a temporal sparsity constraint in the compressed-sensing formulation (*λ*_*t*_ = 0 in equation (1)); a second R1 was performed with a temporal sparsity constraint. More residual noise-like artifacts remained for the conventional 4D flow reconstruction and the R1 without the temporal constraint compared to the R1 with the temporal constraint. In Fig. 4, the reduction of noise was observed in both the magnitude images and also in the velocity images. Conventional 4D flow resulted in sharper images compared to XD flow without the temporal sparsity constraint. With the temporal constraint included, image sharpness was improved over conventional 4D flow (Fig. 3b).

### Respiratory-resolved 4D flow imaging

**Figure 5:**
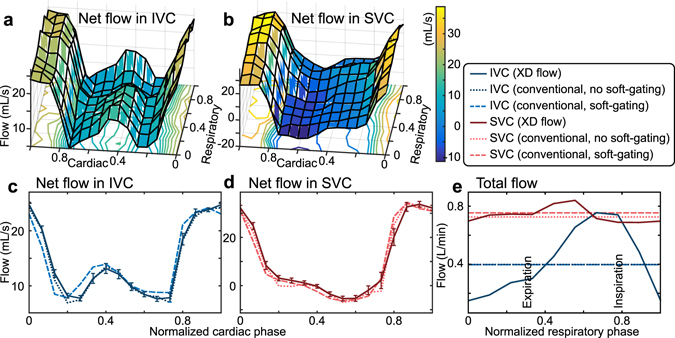
Ferumoxytol-enhanced XD flow reconstruction of a 3-year-old female highlighting the impact of respiration on flow analysis. The net flow through the inferior vena cava (IVC) and the superior vena cava (SVC) are plotted as a function of normalized cardiac and respiratory phase in (**a**) and (**b**). The average net flow across the different respiratory phases is plotted in (**c**) and (**d**) as a function of normalized cardiac phase. Blood flow measured in a conventional 4D flow reconstruction (with and without respiratory soft-gating) of the same dataset is also plotted in (**c**) and (**d**) as dotted and dashed lines. In (**e**), the total flow in the IVC and SVC is plotted as a function of normalized respiratory phase. The total flow is observed to be dependent on the respiratory phase. This feature is ignored in the conventional 4D flow reconstructions (dotted/dashed lines).

With the respiratory-resolved 4D flow (reconstruction R2), respiratory-dependent blood flow can be measured^33, 37^. In Fig. 5, flow in the inferior vena cava (IVC) and the superior vena cava (SVC) were measured as a function of both respiratory phase and cardiac phase in an intubated 3-year-old patient volunteer (Fig. 5a and b). The results were compared with conventional 4D flow where the respiratory-phase dimension and its effects were ignored or suppressed. Computing the respiratory variation of the flow measurements in the XD flow dataset, we observed a standard error of 0.77–3.5 mL/s in the IVC and 0.34–1.0 mL/s in the SVC (Fig. 5c and d). A range of 0.75–0.84 L/min (mean of 0.74 L/min) for the SVC and a range of 0.15–0.69 L/min (mean of 0.44 L/min) for the IVC were observed when computing the total average flow as a function of respiration (Fig. 5e). The maximum flow in the IVC was observed during inspiration — an expected physiologic effect. From the conventional 4D flow reconstruction, a total flow of 0.40 L/min (without respiratory soft-gating) and 0.40 L/min (with respiratory soft-gating) in the IVC and a total flow of 0.72 L/min (without respiratory soft-gating) and 0.75 L/min (with respiratory soft-gating) were measured. In Fig. 3, XD flow resulted in sharper images compared to conventional 4D flow.

### Comprehensive cardiopulmonary MRI exam

**Figure 6:**
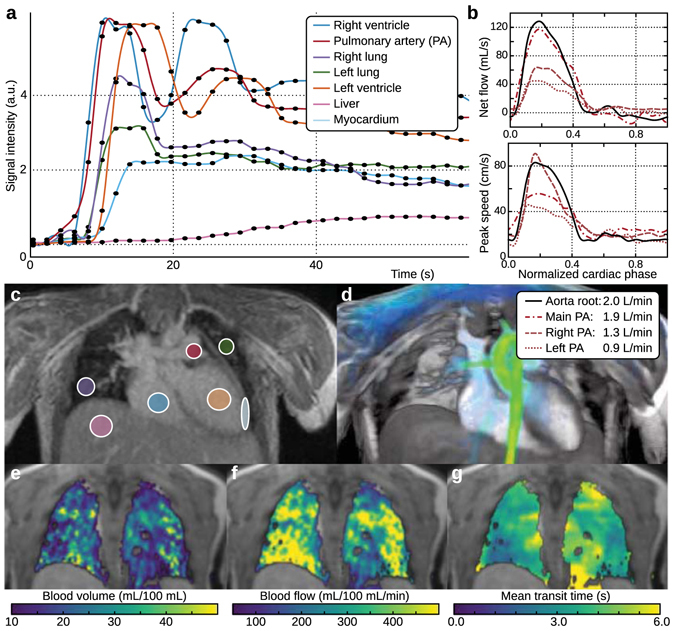
Dynamic-contrast-enhancement/perfusion reconstruction with XD flow of a 2-year-old male with the administration of gadobutrol. Contrast dynamics (2-s temporal resolution) of specific tissues are depicted in (**a**) with the corresponding ROI drawn in (**c**). The same dataset can also be reconstructed to depict flow information as plotted in (**b**) and visualized in (**d**). In (**a**), the initial enhancement of the right ventricle can be seen with the initial peak. The contrast recirculating through the system can be observed with the secondary peak. Pulmonary enhancement can be seen with the ROIs drawn on the right and left lungs. Pulmonary perfusion maps are displayed as pulmonary blood volume (**e**), pulmonary blood flow (**f**), and mean transit time (**g**). The enhancement of the lungs is after the enhancement of the right ventricle and pulmonary artery.

**Figure 7:**
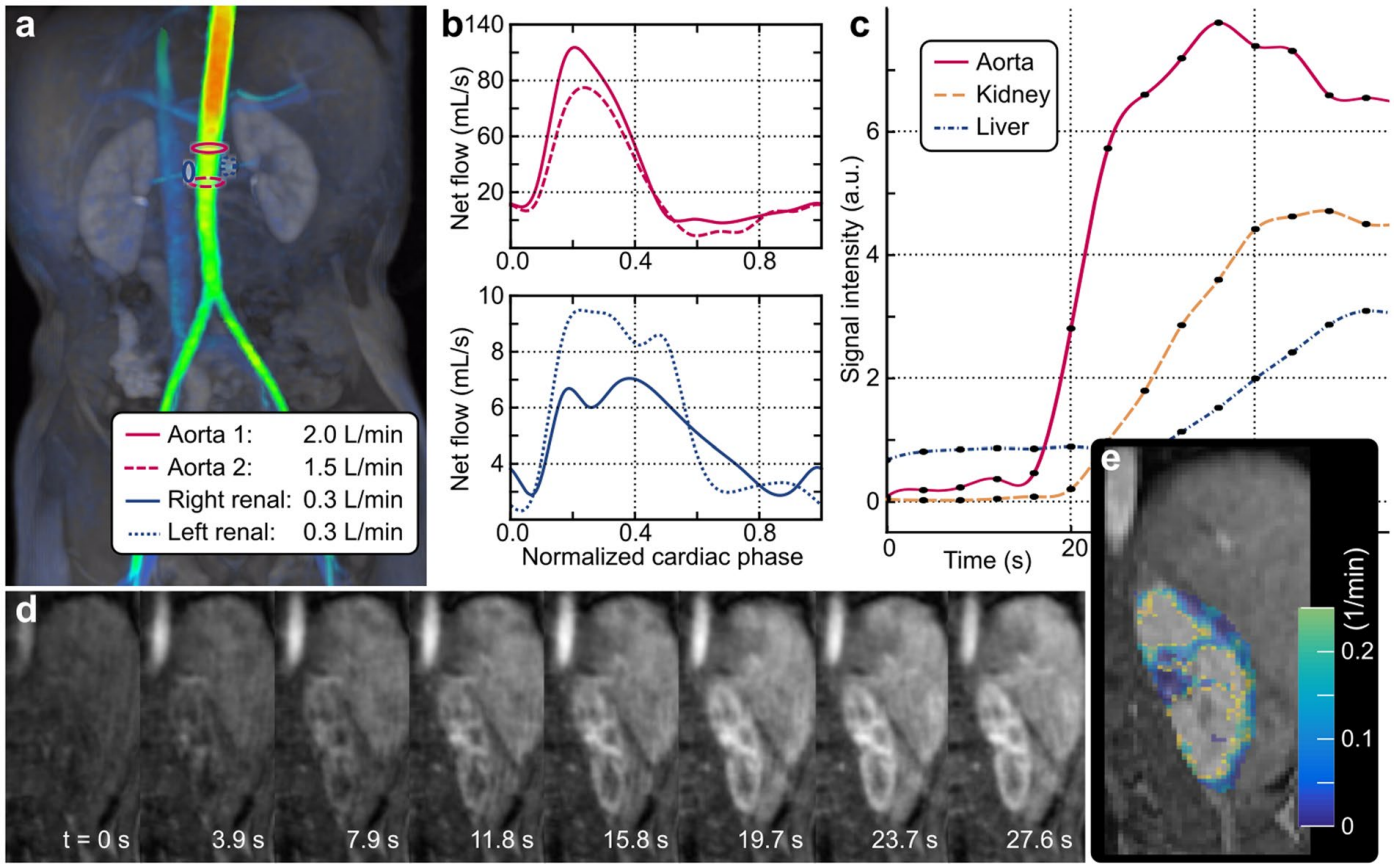
XD flow imaging using reconstruction R3 in the abdomen of an 8-year-old male with gadobutrol administration to enable comprehensive kidney assessment. Blood flow velocities are visualized with a surface rendering in (**a**) and net flow plotted in (**b**). The blood flow in the aorta was measured superior (solid red) and inferior (dashed red) to the renal arteries (blue) to demonstrate internal consistencies with flow measurements. The same dataset is reconstructed at a 3.9-s temporal resolution. The signal enhancement curves are plotted in (**c**) and the dynamics are highlighted in the left kidney in (**d**). From this dataset, a glomerular filtration rate map is generated and displayed in (**e**).

The feasibility of using XD flow for assessing contrast dynamics was performed, and the results are shown in Figs 6 and 7. Conventional 4D flow imaging ignores contrast dynamics, and therefore, an additional scan is typically required for dynamic-contrast-enhancement imaging. Using reconstruction R3 of XD flow, a higher-temporal resolution dataset was reconstructed with fewer cardiac phases from the same dataset. In the study of a 2-year-old male with gadobutrol administration, the contrast uptake in the lungs can be seen in the dataset with a temporal resolution of 2 s (Fig. 6a). The contrast-enhancement curves agreed with normal physiology: first enhancement of the right ventricle, followed by the pulmonary artery (PA), then the two lungs, and lastly, the left ventricle. The slower uptake in the liver and myocardium was also observed. Using the signal time curves of the main pulmonary artery as an arterial input function, perfusion maps were generated. For the displayed slice in Fig. 6g, the mean transit time (MTT) of the lungs was estimated to be 4.0 ± 1.3 s (mean standard deviation). The pulmonary blood volume (PBV) and pulmonary blood flow (PBF) were respectively estimated to be 22.2 ± 9.8 mL/100 mL and 359.4 ± 203.2 mL/100 mL/min. These quantities were in agreement with previously reported values for end expiration^38^: from 9 subjects, mean MTT of 4.2 s, PBV of 22 mL/100 mL, and PBF of 316 mL/100 mL/min. The same dataset was reconstructed to assess flow (Fig. 6b). Here, the third temporal bin out of three temporal bins was used to measure blood flow. The flow analysis had good internal agreement: flow in the main PA (1.9 L/min) approximately equals the flow in the aorta root (2.0 L/min), and flow in the main PA approximately equals the total flow through the left PA (0.9 L/min) and the right PA (1.3 L/min). Image sharpness for XD flow was slightly reduced in comparison to conventional 4D flow (Fig. 3d).

The proposed XD flow method can be applied for other clinical applications. As an example, a comprehensive kidney MRI exam was demonstrated in Fig. 7. Here, the third temporal phase out of a total of three was used to measure blood flow (Fig. 7a and b). The flow in the aorta was measured superior and inferior to the renal arteries. Good internal consistencies were observed: flow in the aorta before the renal arteries (2.0 L/min) approximately equals the sum of the flows in the right renal artery (0.3 L/min), left renal artery (0.3 L/min), and aorta after the renal arteries (1.5 L/min). Using reconstruction R3, the same dataset was reconstructed with 5 cardiac phases and 3.9-s temporal bins. The contrast-enhancement curves measured in the aorta, liver, and kidney cortex are plotted in Fig. 7c. A subset of the corresponding magnitude images are displayed in Fig. 7d. From R3, a glomerular filtration rate map was generated (Fig. 7e) with a mean of 0.18 ± 0.14 min^−1^ in the cortex of the left kidney for the displayed slice.

## Discussion

A single rapid imaging test that can be used for flow quantification, cardiac/respiratory function analysis, anatomical assessment, and contrast-enhancement/perfusion evaluation was developed. This comprehensive MRI sequence simplified the cardiac MR protocol and extended it to include kidney or pulmonary function assessment. Furthermore, by generating different reconstructions from the same data acquisition, the post-processing analysis for each feature was automatically registered to each other. This advantage can be exploited to potentially enhance post-processing analysis. For example, the contrast-dynamics for different vessels can be leveraged to aid in differentiating between arteries and veins for better visualization or for more robust automatic vessel segmentation.

In this work, the rapid test of XD flow imaging was developed and applied to several clinical examples. The XD flow technique relied on the adaptation and the combination of three key previous developments. First, a pseudo-random variable-density sampling and radial view-ordering (VDRad) technique^25, 39^ enabled retrospective re-sorting of the data to highlight different states and dimensions. Second, intrinsic Butterfly navigators^25, 40^ provided the motion information needed, with no time penalty, to resolve respiratory motion and/or to suppress motion-induced image artifacts. Given the high temporal rate of the Butterfly navigators cardiac signal can also be extracted^41^. Thus, the entire XD flow acquisition can be performed with no external physiological signal monitoring, further simplifying scan setup. Third, the higher subsampling factors can be mitigated by leveraging the increase in data redundancies in higher-dimensional space with a compressed-sensing reconstruction^29^. Here, separable constraints were applied for each data dimension. The reconstruction can be further improved with more sophisticated models that encompass multiple dimensions, such as the low-rank penalty in the spatial-temporal dimensions^42, 43^. This investigation will be our future work.

Ideally, the different datasets can be generated through one large reconstruction that properly shares the redundant information. However, such a reconstruction results in an optimization with millions of variables (each additional dimension increases the number of variables by approximately a factor of 10), which is currently too time consuming to perform clinically. Here, we compromised and performed three separate reconstructions that were relatively faster and more manageable in terms of required computation. Given the same data acquisition for the different reconstructions, results from one reconstruction can be easily used to initialize or constrain subsequent reconstructions. Each separate reconstruction was also highly parallelizable, and the entire framework can be adapted to use parallel computing and cloud computing for even faster reconstruction times^44^. For a single dataset, a single set of ESPIRiT sensitivity maps^45^ was used for each separate reconstruction to reduce the time and memory needed for computing these maps that model the sensitivity profiles for each element in a coil-receiver array. Reconstruction accuracy can possibly be improved with more accurate ESPIRiT sensitivity maps for every *c*, *r*, *t*, and *f*. However, guaranteeing that sufficient data samples are collected for computing accurate maps at each point becomes increasingly difficult with higher subsampling factors in this multi-dimensional space.

Lifting dimensionality while maintaining scan durations results in highly subsampled datasets, though the increase in dimensionality does not correspond to the increase degrees of freedom. Increase data redundancy allows for the recovery of the increase number of missing samples. Unfortunately, no real gold standard exists to assess the impact of this process on spatial and temporal resolutions. A fully sampled XD flow acquisition will require over an hour of continuous scanning. Phantom studies are an alternative, but compressed-sensing-based methods can leverage the simplicity of artificial models to produce high quality reconstructions. Additionally, reconstruction techniques can be designed to favor one dimension over the others. For example, simple keyhole reconstruction^46, 47^ produces fully sampled datasets at each time point which results in high spatial resolutions at the cost of temporal resolutions. Minimal sharing of information between time points results in highly subsampled datasets with residual aliasing and blurring, but temporal information can be extracted with high temporal resolutions^48^.

As an initial assessment, we compared XD flow with the original soft-gated 4D flow which has been previously validated for structure, flow, and function^19, 25^. All acquisitions were originally optimized for 4D flow, and this setup provided a baseline for comparison when we reconstructed these same acquisitions as XD flow. A major advantage of this approach was that no additional acquisitions or external data were required to assess image quality for the different XD flow reconstructions. Here, we performed this comparison in terms of image sharpness using the gradient entropy metric^49^, and the analysis demonstrated whether spatial resolutions were gained or lost with XD flow. For future work, internal consistency will be essential to indicate the quality of XD flow datasets. These internal consistency checks include blood flow through the aortic root should equal blood flow through the main pulmonary artery, volumetric change in the cardiac ventricles should equal total cardiac blood flow output, and total pulmonary perfusion should equal total blood flow through the pulmonary arteries. The advantage of assessing internal consistency of measurements is that these measurements themselves are critical for diagnosis; in certain applications, the accuracy of these measurements are more important than the apparent image quality.

The techniques discussed can be directly applied to non-Cartesian approaches to flow imaging, such as three-dimensional radial imaging^50^ or stack of spiral^51^. These special k-space trajectories innately provide the properties that VDRad is designed to replicate: robustness to motion and variable-density sampling for compressed sensing. When exploring different k-space sampling trajectories, one must consider the increase in computation time needed for reconstructing non-Cartesian datasets with compressed sensing.

The ability to add a temporal dimension to flow velocity provides a tool for potentially more robust flow imaging of uncooperative patients. An alternative is to prescribe a shortened flow imaging acquisition with reduced resolutions in either the cardiac-phase or the spatial dimensions. However, in such an approach, the acquisition is innately limited to those prescribed resolutions. A higher-resolution imaging sequence that is slightly longer has the potential to produce high-resolution flow images if the patient is cooperative during the acquisition. In the situation of the pediatric patient becoming restless during the scan, XD flow has the ability to still produce clinically useful images by temporally binning the data. Further, the additional data from neighboring temporal bins provide information that can be exploited in the compressed-sensing-based reconstruction. The temporal component can also be exploited by correcting for patient motion between temporal bins and combining the different temporal bins in a final reconstruction. This approach will improve SNR and reduce motion-induced image artifacts.

The ability to quantify blood flow velocities in higher-dimensional space provides a tool for potentially more accurate and reproducible flow quantification. The impact of respiration on cardiac flow velocities has been demonstrated here. If the pediatric patient respiratory pattern changes from one MRI exam to the next or if the pediatric patient is intubated in one exam and not in the other, flow quantification can differ. By enabling the ability to select any arbitrary respiratory phase to investigate flow, more consistency in the state of the patient can possibly be achieved to better measure flow, and its changes in evolving diseases. Respiratory-resolved flow imaging will also provide the tool to investigate how intubation or anesthesia impacts flow measurements and to investigate the relationship between respiratory and cardiac systems.

Though the proposed approach has been migrated into our clinical practice and we have demonstrated its potential to provide more accurate flow quantification and yield additional diagnostic information, a thorough clinical validation for specific disease indications is beyond the scope of this work and will be our focus for future efforts. Here, we propose a framework for XD flow and demonstrate the feasibility with results from a variety of example cases. Further work will be required to assess different aspects of XD flow, including the impact of velocity-encoding gradients on the accuracy of perfusion/DCE analysis and the impact of contrast enhancement and respiration on flow quantification accuracy. This type of validation work is nontrivial even in phantom studies, which do not accurately reflect *in vivo* scans. However, the proposed framework provides a means to improve current 4D flow studies. As a 5–15 min scan, it also has the potential to investigate some of these issues in a clinically-realizable manner. XD flow imaging has been developed to improve image quality and to provide additional information (respiratory, contrast dynamics) for assessment.

## Methods

### Data acquisition

A standard Cartesian 4D flow sequence was used on 1.5T and 3T MRI scanners. This RF-spoiled gradient echo sequence included velocity-encoding gradients that produced non-zero first moments that resulted in linear phases proportional to the velocity. These gradients can be designed using bipolar gradients, but to achieve shorter echo times (TE), these gradients were combined with spatial encoding gradients^52^. Velocity images were computed from the phase difference of images encoded with a different velocity-encoding gradient (with a different first moment). At least 4 different gradient configurations are needed to compute 3D velocities — more than 4 configurations are useful for increasing the signal-to-noise ratio while minimizing phase wraps^53^. For a shorter scan duration, we used the minimum number of 4 configurations.

This standard Cartesian 4D flow sequence was modified to use a variable-density sampling and radial view-ordering technique (VDRad)^39^ to produce unique pseudo-random sampling patterns for each cardiac phase and for each velocity-encoding echo. For MRI, spatial information in (*x*, *y*, *z*) are acquired in the corresponding k-space domain (or frequency domain) as (*k*_*x*_, *k*_*y*_, *k*_*z*_) with *k*_*x*_ fully sampled for Cartesian imaging. With VDRad, (*k*_*y*_, *k*_*z*_)-views on a Cartesian grid were grouped into variable-density spiral spokes. The acquisition of different spiral spokes were ordered according to the golden-ratio angle increment^54^.

VDRad should be optimized for different clinical questions. Here, we focused on resolving pulsatile blood flow dynamics; thus, the acquisition scheme was optimized for cardiac-triggered imaging. This view-ordering design is described in detail in refs 25, 39 and is described briefly here. The VDRad scheme was first used to generate *N*_*c*_ × *N*_*e*_ sampling masks where *N*_*c*_ is the desired number of cardiac phases and *N*_*e*_ is the number of different velocity-encoding echoes. Each individual sampling mask specifies what (*k*_*y*_, *k*_*z*_)-views to collect and in what order. Due to the golden-ratio angle increment ordering, each adjacent mask was complementary to the other. Combining samples from adjacent masks will decrease undersampling reduction factor *R* (Fig. 1b). *R* was adjusted for the receiver coil array, patient size, and gains from compressed sensing. During data acquisition, the (*k*_*y*_, *k*_*z*_)-sample to collect was determined by the corresponding sampling mask of the current velocity-encoding echo and current cardiac phase.

The data acquisition window was extended to include velocity-encoding gradients. These velocity-encoding gradients were repeatedly applied throughout the scan and sampled the same trajectories in k-space. Thus, these gradients were used as intrinsic MR navigators to monitor patient motion with high temporal fidelity^25^. As an adaption of a previous technique^39^, we refer to this approach with the same name of “Butterfly” navigators. Further, with a multi-channel coil receiver array, each element provided localized sensitivity that can be exploited to help extract physiological signals and the time of initial contrast injection. In this work, the velocity was encoded using a minimum echo time (TE) configuration^52^, but any standard velocity-encoding configuration can be used.

The sequence was prescribed to run for 5–10 min. Contrast was intravenously administered either before the sequence or 1–2 min after the sequence was started. See Fig. 1a for a graphical description of the data acquisition. Specific scan parameters are summarized in Table 1.

### Image reconstruction

From one data acquisition, different compressed-sensing-based parallel-imaging reconstructions^21, 24, 29, 55^ were performed with the following optimization problem:

1 $$\hat{m}={\rm{\arg }}\,\mathop{{\rm{\min }}}\limits_{m}\frac{1}{2}{\Vert W(Am-y)\Vert }_{2}^{2}+{\lambda }_{x}{R}_{x}(m)+{\lambda }_{t}{R}_{t}(m)+{\lambda }_{c}{R}_{c}(m)+{\lambda }_{r}{R}_{r}(m\mathrm{).}$$

Matrix *A* models the acquisition process with coil-receiver sensitivity maps, Fourier transform, and subsampling. Sensitivity maps were estimated using the ESPIRiT algorithm^45^ from a calibration k-space region (with a size of 24 × 24 × 24) that was generated by projecting the entire data acquisition for a single flow-encoding echo into a single spatial volume. The generated calibration data was robust to motion effects due to the VDRad sampling design with variable-density sampling and repeated sampling of the k-space center. This calibration strategy has been previously demonstrated for both dynamic-contrast-enhancement imaging^43^ and 4D flow imaging applications^25^.

Matrix *A* transforms the image set *m*, fully resolved XD flow images, to the acquired k-space data *y*. For compressed sensing, regularization functions R_*_(*m*) and regularization parameters λ_*_ penalize non-sparse solutions in the spatial (*x*), temporal (*t*), cardiac-phase (*c*), and respiratory-phase dimensions (*r*). Temporal dimension refers to time over the scan (relative to contrast injection bolus for perfusion). In this work, the ${\ell }_{1}$-norm of the Daubechies D4 wavelet transform was used for *x*, and the ${\ell }_{1}$-norm of the finite difference was used for all other dimensions. Regularization parameters λ_*_ were empirically determined for each reconstruction (R1, R2, and R3) and held constant for subsequent datasets. Single *x*-slices (2D in the spatial) were used to rapidly determine these parameters. The regularization parameters are adjusted based on the sparsity constraint, because different functions for R_*_(*m*) result in different degrees of sparsity and different scaling factors. Specifically, we started with *λ*_*x*_ = 1 × 10^−5^ for the wavelet transform, and we started with *λ*_*t*_ = 0.01, *λ*_*c*_ = 0.01, and *λ*_*r*_ = 0.01 for the finite differences. These parameters were then tuned for each reconstruction type.

The Butterfly signal was used to determine the respiratory phase of each data point and time of contrast injection. Data were binned according to time of acquisition, respiratory phase, and cardiac phase. Matrix *W* weighed the data according to how far the data was from the center of each bin and how much patient motion occurred during the acquisition of that particular data point (see Supplementary Fig. S1). For non-respiratory-resolved imaging, *W* soft-gated the data to suppress image artifacts from respiratory motion^39, 56^. How the values in *W* were computed will be described below.

To enable the application of different regularization constraints in equation (1), the reconstructions were performed using Alternating Direction Method of Multipliers (ADMM)^57, 58^. The benefit of including multiple constraints was demonstrated in Fig. 4. To reduce the dataset size, coil compression^59^ was used to transform the multi-channel data to 6 virtual coils. This number of virtual coils was recommended in ref. 59 for the 32-channel coil, and no noticeable difference in performance was observed for the same number of virtual coils for the 20-channel coil.

### Soft-gating weights

k-Space data were binned based on the (*k*_*y*_, *k*_*z*_) location, the time the data point was acquired, the cardiac phase, and the respiratory phase. Because this binning process discretized time, cardiac phase, and respiratory phase, each data point was weighted by the distance of each point from the center of each bin. This process avoided the need of gridding the data and was straightforward to include in the soft-gating framework of equation (1). In this work, weights were computed using a simple Hanning window.

As an example, the weight for the *n*-th k-space data, placed into the *t*_0_ temporal bin, the *c*_0_ cardiac bin, and the *r*_0_ respiratory bin, was computed as

2 $$w[n,{t}_{0},{c}_{0},{r}_{0}]={w}_{t}[n,{t}_{0}]\times {w}_{c}[n,{c}_{0}]\times {w}_{r}[n,{r}_{0}]\times {w}_{d}[n\mathrm{].}$$

Function *w*_*t*_[*n*, *t*_0_] for the acquisition number *n* was computed as

3 $${w}_{t}[n,{t}_{0}]=\{\begin{array}{ll}0.5+0.5\,\cos \,[\frac{\pi }{{T}_{t}}({t}_{0}-n\times {\rm{TR}})], & |\frac{1}{{T}_{t}}({t}_{0}-n\times {\rm{TR}})|\le 1\\ \mathrm{0,} & {\rm{otherwise}}\end{array}\mathrm{.}$$

The repetition time (TR) specifies the time increment between each data point, and *T*_*t*_ specifies the width of the temporal window. Both quantities have the same time units, e.g. ms. Similarly, functions *w*_*c*_[*n*, *c*_0_] and *w*_*r*_[*n*, *r*_0_] were computed as

4 $${w}_{c}[n,{c}_{0}]=\{\begin{array}{ll}0.5+0.5\,\cos [\pi ({c}_{0}-c[n]\times {N}_{c})], & |{c}_{0}-c[n]\times {N}_{c}|\le 1\\ \mathrm{0,} & {\rm{otherwise}}\,\end{array},$$

and

5 $${w}_{r}[n,{r}_{0}]=\{\begin{array}{ll}0.5+0.5\,\cos [\pi ({r}_{0}-r[n]\times {N}_{r})], & |{r}_{0}-r[n]\times {N}_{r}|\le 1\\ \mathrm{0,} & {\rm{otherwise}}\end{array}\mathrm{.}$$

**Figure 8:**
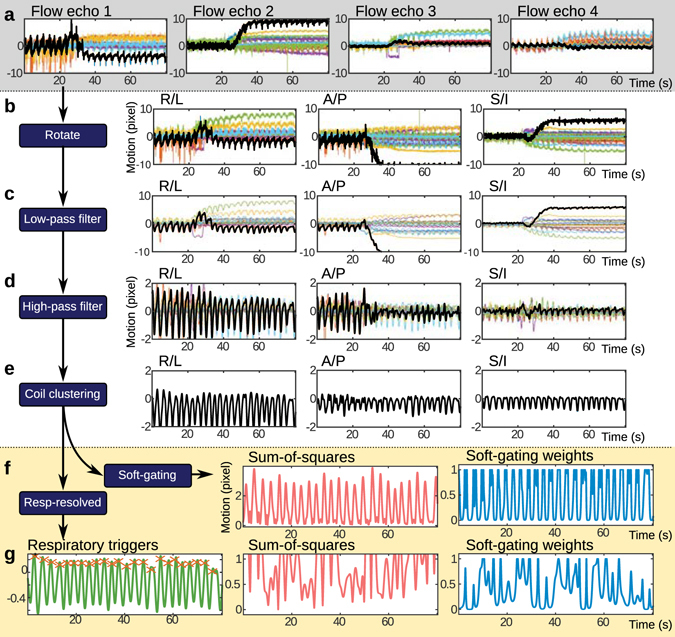
Processing of motion estimates for physiological signal monitoring. From the 4 different velocity-encoded echoes (4 flow echoes), 4 different directions of motion are measured using the Butterfly navigators and are plotted in terms of image pixels in (**a**). Each color represents a different channel from a 32-channel cardiac coil receiver. In (**b**), these estimates are rotated to the conventional right/left (R/L), anterior/posterior (A/P) and superior/inferior (S/I) directions. In (**c**), a low-pass filter tuned with a cut-off frequency based on the recorded cardiac rate is used to remove the cardiac signal and noise. In (**d**), a high-pass filter is applied to remove the low-frequency drifting that is partially attributed to the contrast administration. In (**e**), a coil-clustering algorithm is used to extract the dominate motion. Afterwards, the motion estimates can be directly used to compute soft-gating weights (**f**) or further processed for respiratory-resolved imaging (**g**). For respiratory-resolved imaging, the respiratory trigger points can be located and used to determine respiratory phases. Soft-gating weights based on residual patient motion from changing respiratory depth or bulk patient motion can then be computed.

Functions *c*[*n*] and *r*[*n*] respectively specify the cardiac phase and the respiratory phase of the *n*-th acquisition; these were normalized between 0 and 1. The number of desired cardiac phases and the number of desired respiratory phases are represented as *N*_*c*_ and *N*_*r*_. Function *c*[*n*] was derived from the scanner’s cardiac triggering system. Function *r*[*n*] was computed from the respiratory motion derived from the Butterfly navigators. Function *w*_*d*_[*n*] considered other types of patient motion and was also derived from the Butterfly navigators. The processing of Butterfly navigators will be described in the subsequent paragraphs and is summarized in Fig. 8.

In order to extract motion from the multi-channel motion, the following steps were performed. First, the motion was estimated from the raw Butterfly k-space data using the method detailed in ref. 40. Second, each motion estimate from each coil element was filtered using a low-pass filter with a cut-off frequency of 95% the measured heart rate. This process removed both high-frequency noise and cardiac motion from the estimates. The 95% factor accounted for possible variations in the heart rate. A high-pass filter with a cut-off frequency of 0.1 Hz was also applied to remove drifts in the motion estimates. For contrast-enhanced studies, contrast changes induced a drift in the estimates. Third, a single motion estimate **d**_0_[*n*] was extracted from the multi-channel data using an automated coil clustering algorithm^41^. For the Butterfly navigator, three-dimensional motion was measured; therefore, **d**_0_[*n*] is a 3-element vector. Finally, the norm of **d**_0_[*n*] was computed and then normalized by its standard deviation:

6 $${d}_{1}[n]=\frac{\Vert {{\bf{d}}}_{0}[n]\Vert }{{\rm{std}}(\Vert {{\bf{d}}}_{0}[n]\Vert )}\mathrm{.}$$

For reconstructions R1 and R2, the respiratory motion was not resolved. Instead, soft-gating was used to suppress artifacts from respiratory motion. In this case, *w*_*r*_[*n*, *r*_0_] from equation (5) was set to 1 for all values of *n* and *r*_0_, and *w*_*d*_[*n*] was computed as

7 $${w}_{d}[n]=\{\begin{array}{ll}{e}^{-\alpha (d[n]-\beta )}, & d[n] > \beta \\ \mathrm{1,} & {\rm{otherwise}}\end{array}\mathrm{.}$$

Equation (7) is based on refs 40, 56. A histogram analysis was performed on *d*_1_[*n*], and the motion was centered on the motion position with the largest bin: *d*[*n*] = *d*_1_[*n*] − *d*_*ref*_. Constant *β* specified the cutoff value where anything below this value was accepted as uncorrupted data with weights of 1. Any values below *β* were exponentially weighted down by factor *α*. The values of these constants were based on reported literature^40^: *α* = 1.0 and *β* = 0.25.

For the respiratory-resolved reconstruction R3, *d*[*n*] was further processed. With the cardiac signal suppressed with the low-pass filter, the dominant signal in the motion estimate was respiratory motion. Thus, *d*[*n*] was processed to determine the triggers for respiration, and a peak finding algorithm was used to extract respiratory triggers. These respiratory triggers were used to derive the respiratory-phase function *r*[*n*], normalized between 0 and 1. To consider unaccounted patient motion, *d*[*n*] can be further filtered with a low-pass filter tuned with a cut-off frequency of 95% the derived respiratory rate. This final *d*[*n*] can then be centered based on the histogram analysis and finally used to compute *w*_*d*_[*n*] in equation (7). Given the extremely high subsampling factors when constructing the dataset in higher-dimensional space, we did not incorporate these respiratory-filtered soft-gating weights when resolving respiratory motion.

The VDRad design and the discretization of the multi-dimensional space resulted in multiple measurements for the same (*k*_*x*_, *k*_*y*_, *k*_*z*_, *t*, *c*, *r*) locations. To simplify the formulation, these repeated samples were replaced by the weighted average of the samples. The weights in this weighted average were the square of the soft-gating weights, (*w*[*n*, *t*_0_, *c*_0_, *r*_0_])^2^. The soft-gating weights were then replaced by the square root of the sum-of-squares of these weights. As described in more detail in the Supplementary Methods, this modification did not impact results, enabled the use of the original solver, and minimized memory needed for computation.

### Post-processing analysis

For flow quantification, phase-contrast images were first corrected for Maxwell phase errors^60^ and gradient nonlinearity^61^. Background phase errors were estimated from the static tissue using a third-order polynomial model and were subtracted from the velocity images. Different region-of-interests (ROI’s) were drawn to quantify blood flow velocities. Post-processing software (Arterys, San Francisco, California, USA) was used to assist in this image analysis. Additionally, standard deviation of the velocity measurements was derived. The standard deviation was calculated based on velocities of the static tissue. Because the compressed-sensing formulation with total variation reduces variance in the cardiac-phase dimension, a single cardiac phase (normalized cardiac phase of 0.75) was used for the standard deviation calculations.

The resulting volumetric time-series magnitude images from R3 reconstructions were T1-weighted and provided the source data for pulmonary perfusion quantification^62–64^. An arterial input function was estimated from the main pulmonary artery. For each image pixel, the magnitude time series images were de-convolved by this input function using truncated singular value decomposition^65^. Pulmonary blood volume, pulmonary blood flow, and mean transit time were then derived from the resulting signals. An open-source OsiriX plug-in was used for this analysis^66^.

The time series images with dynamic-contrast-enhancement can also be used to assess kidney function using pharmacokinetic modeling. For the post-processing analysis of the kidneys, a three-compartment model^67^ was used to calculate the glomerular filtration rate (GFR). An ROI was drawn in the aorta to estimate the concentration time curves of the arterial input function. Specific details of the GFR calculations can be found in ref. 48. The focus of this study was to evaluate the feasibility of XD flow. Thus, correction for signal concentration linearity was not performed for either the pulmonary perfusion or kidney function analysis.

Image sharpness was quantified using the gradient entropy metric where lower values corresponded to sharper images^49^. The spatial resolution of the final image reconstruction was retrospectively lowered, and the metric was computed as a function of image resolution. This metric should ideally be monotonically increasing as a function of pixel size: lower resolutions (or larger pixel sizes) should result in reduced sharpness (or higher gradient entropy values). The true underlying spatial resolution can be considered as the image resolution with the minimum gradient entropy value. To provide an initial assessment of image quality, XD flow was compared with the original soft-gated 4D flow acquisition since the data acquisition was optimized for conventional 4D flow and this 4D flow has been extensively validated^19, 25^. For volumetric Cartesian imaging, k-space subsampling was performed in the (*k*_*y*_, *k*_*z*_)-plane; thus, subsampling impacts spatial resolution in only the (*y*, *z*)-plane. As a result, the image sharpness analysis was simplified by selecting only the center *x*-slice.

### Code availability

Reconstruction was implemented in MATLAB and C/C++ using Berkeley Advanced Reconstruction Toolbox (BART)^68, 69^. This toolbox can be found at http://mrirecon.github.io/bart.

## Electronic supplementary material

Supplementary Materials
