# Supplementary material for: Comprehensive Multi-Dimensional MRI for the Simultaneous Assessment of Cardiopulmonary Anatomy and Physiology
Source: Sci Rep. 2017 Jul 13;7:5330. doi: 10.1038/s41598-017-04676-8 (PMC5509743; doi:10.1038/s41598-017-04676-8)
Supplement: Supplementary file 1 — Supplementary Materials [file 41598_2017_4676_MOESM1_ESM.pdf]

Comprehensive Multi-Dimensional MRI for the Simultaneous Assessment of Cardiopulmonary Anatomy and Physiology

Joseph Y. Cheng[1,*], **Tao Zhang**[2], **Marcus T. Alley**[1], **Martin Uecker**[3,4], **Michael Lustig**[5], **John M. Pauly**[6], **and Shreyas S. Vasanawala**[1]

[1]Stanford University, Department of Radiology, Stanford, California, USA
[2]GE Healthcare, Houston, Texas, USA
[3]German Center for Cardiovascular Research, Partner Site, Göttingen, Germany
[4]University Medical Center, Göttingen, Department of Diagnostic and Interventional Radiology, Germany
[5]University of California, Berkeley, Department of Electrical Engineering and Computer Sciences, Berkeley, California, USA
[6]Stanford University, Department of Electrical Engineering, Stanford, California, USA
[*]jycheng@stanford.edu

ABSTRACT

Diagnostic testing often assesses the cardiovascular or respiratory systems in isolation, ignoring the major pathophysiologic interactions between the systems in many diseases. When both systems are assessed currently, multiple different modalities are utilized in costly fashion with burdensome logistics and decreased accessibility. Thus, we have developed a new acquisition and reconstruction paradigm using the flexibility of magnetic resonance imaging (MRI) to enable a comprehensive exam from a single 5–15 min scan. We constructed a compressive-sensing approach to pseudo-randomly acquire highly undersampled, multi-dimensionally-encoded and time-stamped data from which we reconstruct entire volumetric cardiac and respiratory motion phases, contrast-agent dynamics, and blood flow velocity fields. The proposed method, named XD flow, is demonstrated for (a) evaluating congenital heart disease, where the impact of bulk motion is reduced in a non-sedated neonatal patient and (b) where the observation of the impact of respiration on flow is necessary for diagnostics; (c) cardiopulmonary imaging, where cardiovascular flow, function, and anatomy information is needed along with pulmonary perfusion quantification; and finally in (d) renal function imaging, where blood velocities and glomerular filtration rates are simultaneously measured, which highlights the generality of the technique. XD flow has the ability to improve quantification and to provide additional data for patient diagnosis for comprehensive evaluations.

Supplementary Materials

Supplementary Methods: Weighted reconstruction with repeated data points

When constructing the data matrix from acquired k-space data, more than one data point can correspond to the same k-space location, temporal bin, cardiac phase, and respiratory phase. The repeated data point can be the result of the acquisition design: in the variable-density sampling and radial view-ordering (VDRad) scheme for Cartesian imaging, increased motion-robustness is achieved by re-sampling the k-space center. The repeated data point can also be the result of discretizing the continuous dimensions of time, cardiac phase, and respiratory phase. These duplicate data points can be included in the reconstruction with minimal modification to the optimization reconstruction problem in equation 1. For simplicity, we will consider data points in one-dimensional space. The concepts are easily extended to higher dimensions.

Let y be an M-element vector where each element corresponds to a measurement of an element in x, an N-element vector. Since y contains repeated measurements of x, the i-th element in x (denoted as x_i) may correspond to multiple elements in y. Let $S[i]$ be the set of all indices of y that corresponds to x_i. Weighting w_j is derived based on the confidence in the measurement of the j-th element of y, denoted as y_j. These weights can also be represented as a diagonal matrix W of size $M \times M$. The j-th row and j-th column of W is denoted as $W_{jj} = w_j$.

Though x can be estimated easily as a weighted average of elements in y, the recovery of x is formulated here as an optimization problem. In this way, the results can be easily applied to equation 1. As a least-squares minimization problem, the

recovery of x as $\hat{x}$ can be written as:

$$\hat{x} = \arg\min_x \|W(Dx - y)\|_2^2 \tag{S1}$$
$$= \arg\min_x x^T D^T W^T W D x - 2x^T D^T W^T W y + y^T W^T W y. \tag{S2}$$

Since W is a diagonal matrix, $W^T W$ is also a diagonal matrix of size $M \times M$ where $(W^T W)_{jj} = w_j^2$. Matrix D maps each element of x to y. Matrix D^T takes each element of y, maps them back to the corresponding locations in x, and sums the repeated measurements.

In the first component of equation S2, $D^T W^T W D$ becomes a diagonal matrix of size $N \times N$ where the nonzero elements are

$$\left(D^T W^T W D\right)_{ii} = \sum_{j \in S[i]} w_j^2. \tag{S3}$$

In the second component of equation S2, $D^T W^T y$ becomes an N-element vector where

$$(D^T W^T W y)_i = \sum_{j \in S[i]} w_j^2 y_j. \tag{S4}$$

Lastly,

$$y^T W^T W y = \sum_j w_j^2 y_j^2. \tag{S5}$$

Let diagonal matrix $\bar{W}$ of size $N \times N$ be defined with the nonzero elements:

$$\bar{W}_{ii} = \sqrt{\sum_{j \in S[i]} w_j^2}. \tag{S6}$$

Additionally, let the i-th element in the N-element vector $\bar{y}$ be defined as

$$\bar{y}_i = \left(\sum_{j \in S[i]} w_j^2 y_j\right) / \left(\sum_{j \in S[i]} w_j^2\right). \tag{S7}$$

Then, the quantities of equations S3–S5 can be rewritten in terms of $\bar{W}$ and $\bar{y}$:

$$\left(D^T W^T W D\right)_{ii} = \sum_{j \in S[i]} w_j^2 = \left(\bar{W}^T \bar{W}\right) \tag{S8}$$
$$\left(D^T W^T W y\right)_i = \sum_{j \in S[i]} w_j^2 y_j = \left(\bar{W}^T \bar{W} \bar{y}\right) \tag{S9}$$
$$y^T W^T W y = \sum_j w_j^2 y_j^2 = \bar{y}^T \bar{W}^T \bar{W} \bar{y} + C. \tag{S10}$$

Equation S10 has a constant C that is in terms of w_i and y_i, and it is independent from the unknown vector x. Thus, C can be ignored in the optimization problem, and $\hat{x}$ can be estimated with the following equation:

$$\hat{x} = \arg\min_x \|\bar{W}(x - \bar{y})\|_2^2. \tag{S11}$$

Solving either equation S2 or equation S11 will yield exactly the same solution.

Equations S6 and S7 are directly applied to the proposed equation 1. In equation 1, the diagonal entries in $\bar{W}$ are the square-root of the sum of squares of corresponding soft-gating weights. The acquired k-space vector y of equation 1 is replaced by a weighted average of the repeated data samples with weights corresponding to the square of the soft-gating weights. Typically, M is larger than N since the acquisition repeats samples. Thus, this substitution avoids expanding the data to larger vectors and matrices. This formulation takes into account the duplicate samples without modifying the underlying solver and reduces computation memory.

	#S1 (Supplementary Fig. 1)
Age	3 years
Gender	F
Heart rate	94 bpm
TE/TR	1.8 ms / 3.8 ms
Resolution	(1.1, 1.1, 2.0) mm
Bandwidth	± 83.33 kHz
VENC	250 cm/s
Coil	32ch cardiac
Contrast	Gadobutrol
Scan time	6:13 min
Scanner	3T (GE MR750)

Supplementary Table S1. Summary of scan parameters for supplementary figure.

		t = 0:00–2:04 min	2:04–4:09 min	4:09–6:13 min	
#S1 (Supplementary Fig. 1)	Conventional (PI only)		(±11.1, ±10.7, ±10.7) cm/2		
	Nearest-neighbor (PI only)	(±23.4, ±28.7, ±27.8)	(±11.4, ±12.1, ±11.6)	(±10.2, ±9.4, ±9.5)	
	Weighted (PI only)	(±17.5, ±20.2, ±19.8)	(±9.6, ±9.9, ±10.3)	(±8.5, ±8.9, ±9.1)	
	Conventional (PI & CS)		(±6.5, ±6.2, ±6.5)		
	Nearest-neighbor (PI & CS)	(±9.3, ±10.8, ±9.3)	(±5.3, ±4.9, ±5.4)	(±5.1, ±4.8, ±5.4)	
	Weighted (PI & CS)	(±8.1, ±8.8, ±8.3)	(±4.9, ±4.5, ±5.0)	(±4.7, ±4.4, ±5.0)	
		t = 0:00–2:39 min	2:39–5:17 min	5:17–8:56 min	8:56–10:34 min
#1 (Fig. 2)	Conventional		(±6.2, ±6.2, ±6.1)		
	XD flow	(±4.8, ±4.7, ±4.6)	(±4.7, ±4.6, ±4.5)	(±4.7, ±4.6, ±4.5)	(±5.2, ±5.1, ±5.0)
		t = 0:00–2:48 min	2:48–5:35 min	5:35–8:23 min	
#2 (Fig. 4)	Conventional		(±11.2, ±11.3, ±11.4)		
	XD flow (no temporal TV)	(±16.0, ±15.6, ±15.9)	(±11.8, ±11.8, ±12.0)	(±11.0, ±11.0, ±11.2)	
	XD flow (w/ temporal TV)	(±12.1, ±11.5, ±12.0)	(±8.3, ±8.3, ±8.4)	(±8.1, ±8.1, ±8.2)	

Supplementary Table S2. Standard deviation of velocity measurements in cm/s for the normalized cardiac phase of 0.75 listed as (right/left, anterior/posterior, superior/inferior) where PI = parallel imaging and CS = compressed sensing.

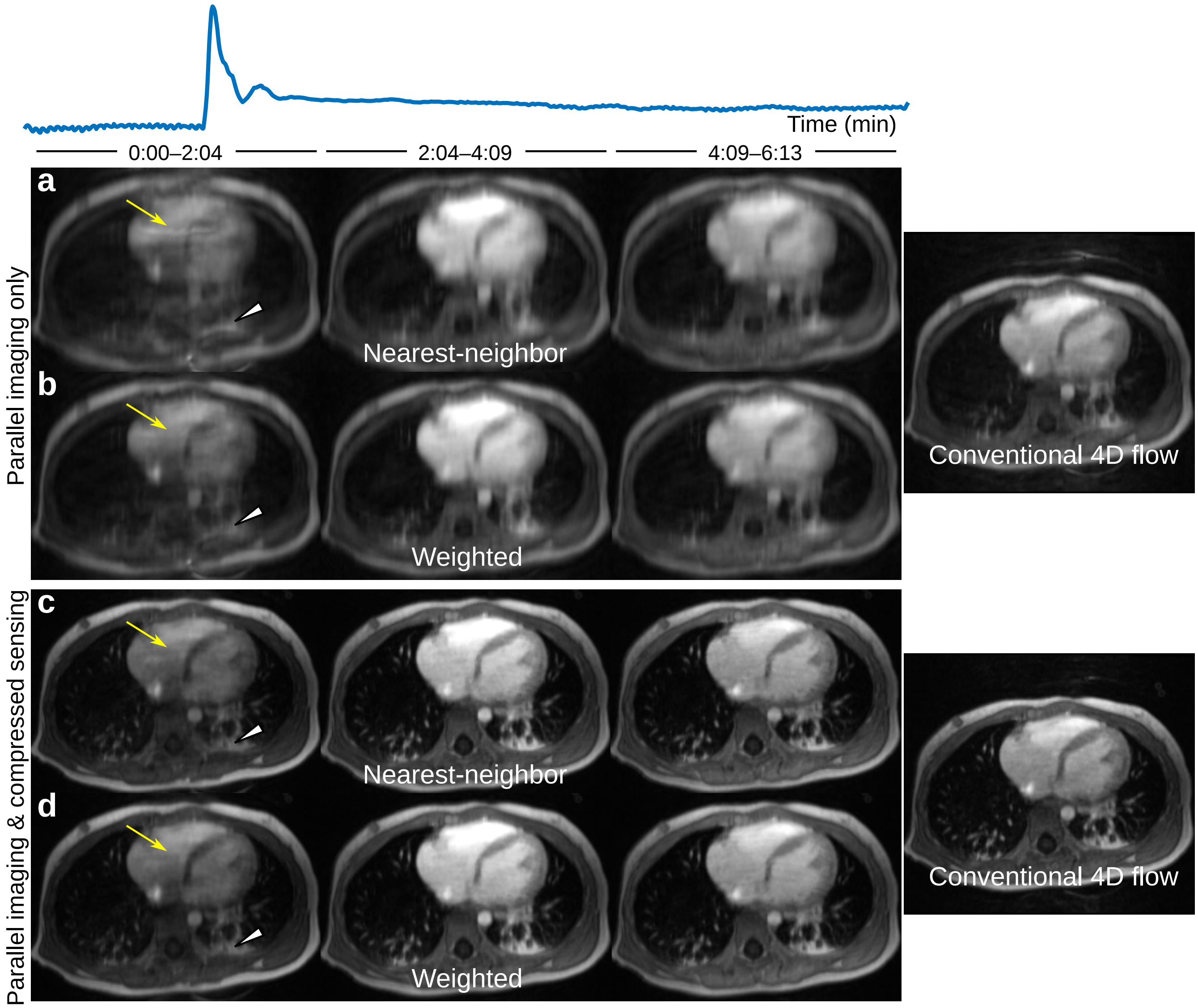

Supplementary Figure S1. Impact of weighting with data binning for XD flow imaging. Gadobutrol was administered during the data acquisition of the 3-year-old female. The dataset was reconstructed with 20 cardiac phases and flow information. The data were divided into 3 temporal bins for the XD flow reconstruction (left) and a single temporal bin for the conventional 4D flow reconstruction (right). For **(a)** and **(c)**, data were binned based on the nearest-neighbor bin. In **(b)** and **(d)**, a weighted reconstruction was used with weights computed based on the distance of each data point to the center of each bin. The data inconsistencies from the contrast dynamics are emphasized in the highly-accelerated parallel-imaging-only reconstruction in **(a)** and **(b)** where residual artifacts are most apparent in **(a)** (yellow arrow and white arrowhead). This can be best seen in the first phase during which the contrast was administered. The de-noising effect from the compressed sensing reconstruction in **(c)** and **(d)** is able to remove much of the artifacts from data inconsistencies. Reduction of the apparent noise in **(d)** is attributed to reducing data inconsistencies.
